# In vitro propagation of bulblets and LC–MS/MS analysis of isosteroidal alkaloids in tissue culture derived materials of Chinese medicinal herb *Fritillaria cirrhosa* D. Don

**DOI:** 10.1186/s40529-020-00286-2

**Published:** 2020-03-24

**Authors:** Hung-Chi Chang, Hui-Min Xie, Maw-Rong Lee, Chiu-Ying Lin, Mei-Kuen Yip, Dinesh Chandra Agrawal, Hsin-Sheng Tsay

**Affiliations:** 1 Department of Golden-Ager Industry Management, Chaoyang University of Technology, Taichung, 413310 Taiwan; 2 King To Nin Jiom Medicine Maf. (Taiwan) Co. Ltd, Wenming 2nd Street, Taoyuan, 33383 Taiwan; 3 Department of Chemistry, National Chung-Hsing University, Taichung, 40227 Taiwan; 4 Department of Applied Chemistry, Chaoyang University of Technology, No.168, Gifong E. Rd, Taichung, 413310 Taiwan; 5 Royal Base Corporation, Changhua, 52446 Taiwan

**Keywords:** Bulblets, *Fritillaria cirrhosa*, Medicinal herb, Peimissine, Tissue culture, Verticine, Verticinone

## Abstract

**Background:**

*Fritillaria cirrhosa,* an important Chinese medicinal herb, is a Class-III protected and highly exploited species by pharmaceutical industry. Dwindling wild populations of species are unable to meet market demand. Therefore, this study was carried out to develop an in vitro propagation method for bulblet production. Also, the study aimed to carry out LC–MS/MS analysis of tissue culture-derived bulblets and callus for the presence of isosteroidal alkaloids (peimissine, verticine, and verticinone), and compare its quantities with commercially available crude drug samples.

**Results:**

In vitro seed germination (91%) of *F. cirrhosa* was achieved on Murashige and Skoog’s basal medium (MSBM) supplemented with 6-benzylaminopurine (1 mg L^−1^) and α-naphthalene-acetic-acid (0.4 mg L^−1^). On transfer of germinated seeds from Petri-dishes to glass bottles containing hormone-free MSBM, 37.5% of seedlings developed bulblets after 3 months of incubation. Regeneration and multiplication of bulblets were achieved by culture of transverse sections of bulblets on 1/2 X MSBM. By repeated subcultures at an interval of 2 months, 3072 bulblets weighing 1270 g could be produced at the end of 5th subculture. LC–MS/MS analysis showed a significant presence of peimissine in in vitro bulblets while callus incubated in the dark showed presence of peimissine and verticine.

**Conclusion:**

The study reports an efficient in vitro propagation method of bulblets production of *F. cirrhosa* and presence of some isosteroidal alkaloids in tissue culture-derived bulblets and callus. The study could be of immense help in production of *F. cirrhosa* bulblets and callus under laboratory conditions round the year. Also, these results can be used further to investigate production of isosteroidal alkaloids in bioreactors at commercial scale using liquid and cell suspension cultures. Thus, we not only can reduce our dependence on collections from natural habitats, but also can help in in situ conservation of this important species.

## Background

The bulbs of various species of genus *Fritillaria* (family Liliaceae) also known as ‘Chuanbeimu’ in Chinese have been used as an antitussive and expectorant in traditional Chinese medicine (TCM) for centuries (The State Pharmacopoeia Commission of P. R. China 2015). So far, about 130 species of *Fritillaria* have been identified worldwide (Lin et al. 2001) which are distributed in the temperate regions of the Northern Hemisphere, mainly in Central Asia and the Mediterranean region. All species of *Fritillaria* are geophytic perennials and bulbiferous. The bulbs are composed of a few fleshy, farinaceous scales, often covered with a translucent tunic (Chen and Mordak 2000).

According to a report, approximately 140 compounds have been isolated from 35 *Fritillaria* species and the majority belongs to isosteroidal alkaloids (72.7%) followed by steroidal alkaloids (11.5%) and non-alkaloids (15.8%), respectively (Lin et al. 2001). Some non-alkaloid constituents containing saponin, terpenoids, steroids, succinic acid, thymidine, adenosine in different *Fritillaria* species have also been identified (Ruan et al. 2002). Chemical and pharmacological studies on ‘Chuanbeimu’ conducted by various researchers have demonstrated that the major biologically active ingredients to relieve cough in the bulb are alkaloids with their types and contents varying in different *Fritillaria* species (Li et al. 2006, 2009). The HPLC–UV analysis of five major pharmacologically active alkaloids of three commonly used *Fritillaria* species showed that the amounts and types of alkaloids varied among them (Ding et al. 1996; Lin et al. 2001). Therefore, quality control of these active principles in herbal ‘Beimu’ is very important to ensure its safe and effective clinical use (Lin et al. 2001).

*Fritillaria cirrhosa* is a perennial, bulbiferous medicinal herb. The species grows at altitudes between 2700 and 4600 m in mountains of Bhutan, South-western China (Chen and Mordak 2000), north-eastern India (Bharali and Khan 2011), and Nepal (Pyakurel and Baniya 2011). The excessive collection of *F. cirrhosa* bulbs from natural habitats by pharmaceutical industry in China has made the species vulnerable and it is now classified as a Class III protected species (Zhang et al. 2010). The importance of *F. cirrhosa* can be gauged from the fact that at present there are about 210 products in the market, extensively used in cough syrups (Cunningham et al. 2018). *Fritillaria* species in general demonstrate very low vegetative propagation in nature, therefore, mass production and cultivation is a serious problem (Carasso et al. 2011). While, the demand of *F. cirrhosa* bulbs is ever increasing, low germination rate, slow plant growth, and restricted growth conditions have posed serious limitation in large scale cultivation of this species. Also, propagation of *Fritillaria* by seed has very little value of practical application because the growth of seedlings is too weak and the development of bulbs is extremely slow and takes about 5–6 years to grow into an apparent size (Ruan et al. 2017).

Petric et al. (2012) in their review article have summarized in vitro morphogenesis studies in different species of *Fritillaria* by various researchers. Bulb was found to be the most widespread explant used for in vitro studies. Explants such as immature embryo and bulb scales in *F. alburyana* and *F. whittallii* have also been reported (Özcan et al. 2007). Gao et al. (1999) demonstrated that in case of *F. unibracteata,* growth rate of bulbs could be increased to 30–50 times compare to natural conditions and it was possible to subculture bulbs for a long time. Paek and Murthy (2002) demonstrated bulblet regeneration in *F. thunbergii* from bulb scale sections at different concentrations of cytokinins and NAA. In China, Wang et al. (2010a, b) carried out limited in vitro regeneration studies on *F. cirrhosa* and callus induction, but further bulblet production and analysis of isosteroidal alkaloids was lacking. To the best of authors’ knowledge, production of *F. cirrhosa* bulblets under culture conditions, and LC–MS/MS analysis of *F. cirrhosa* bulblets and callus have not been reported so far.

**Fig. 1:**
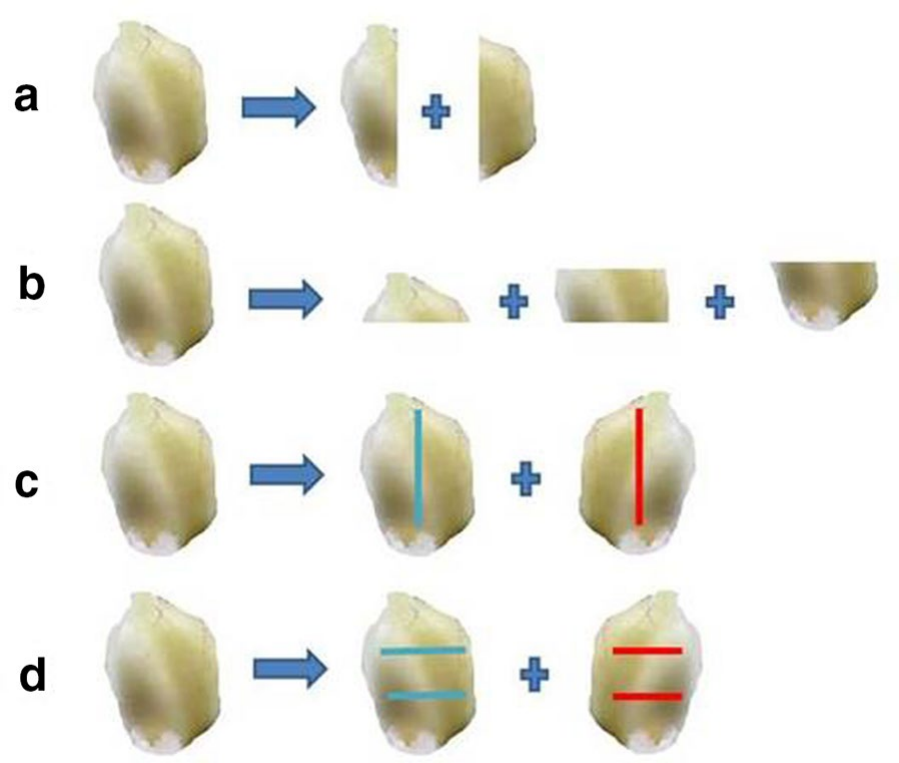
*F. cirrhosa*: Four different sections/incisions **a**: longitudinal section; **b**: transverse section; **c**: longitudinal incision; **d**: transverse incision

In the present study, we established aseptic seedlings by in vitro seed germination, and investigated regeneration and proliferation of bulblets under culture conditions using four different bulblet sections and incisions as shown in Fig. 1a, b, c, d. Also, LC–MS/MS analyses of tissue culture derived bulblets, callus, and commercially available market crude drug sample were carried out. The results obtained could be of immense help in production of *F. cirrhosa* bulblets under laboratory conditions throughout the year and could reduce our dependence on collections from wild. The study also can help in in situ conservation of this important species.

## Results

**Fig. 2:**
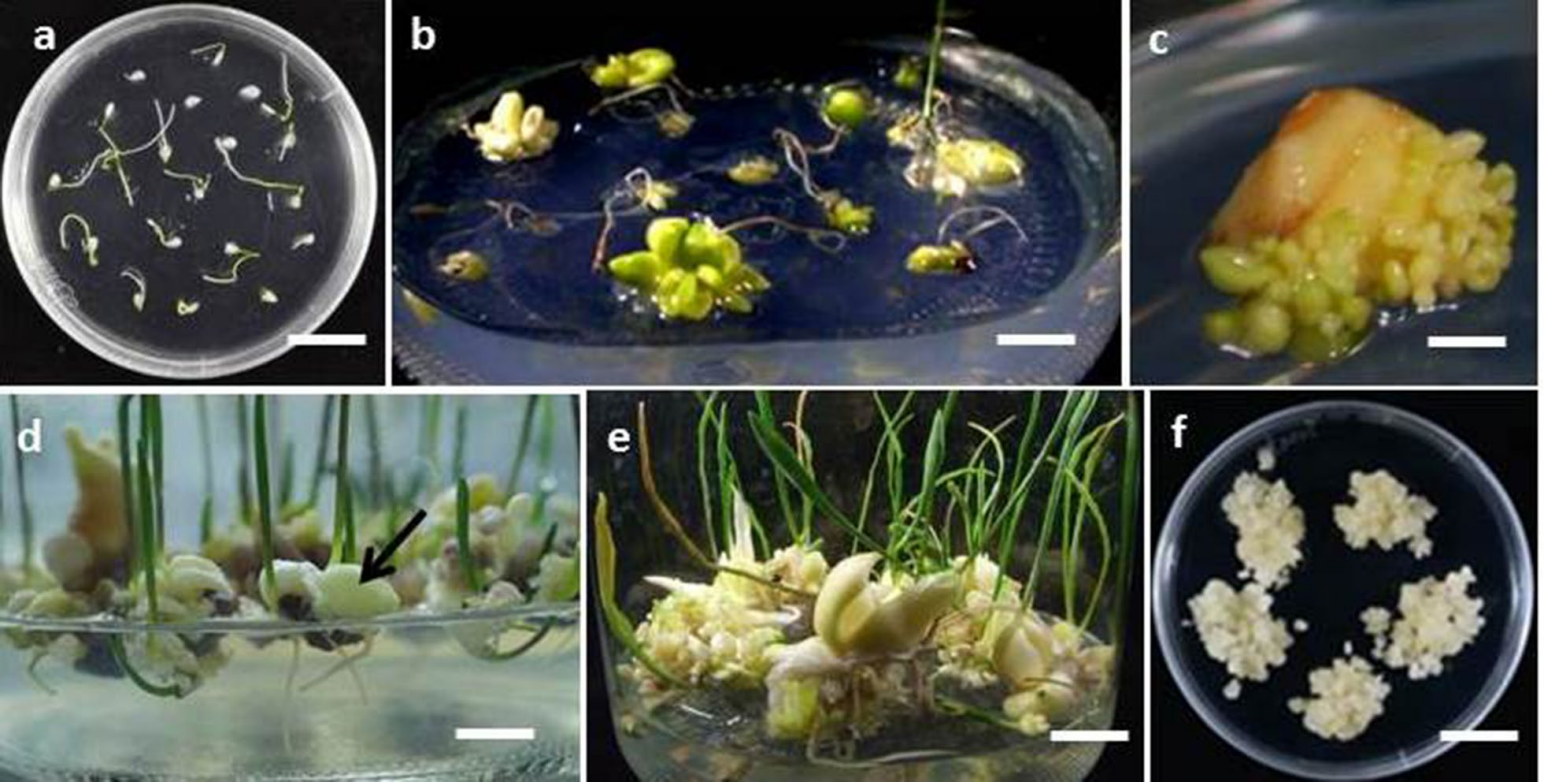
*F. cirrhosa*: **a**: In vitro seed germination on MS basal medium supplemented with BA (1.0 mg L^−1^), NAA (0.4 mg L^−1^) after incubation for 20 days (bar = 1.8 cm). **b**: Bulblet and callus formation in 4 month old seedlings growing on 1/2X MS basal medium supplemented with sucrose (2.5%), GPP (0.4%) (bar = 1.24 cm). **c**, **d**, **e**: Bulblet regeneration from bulb sections after 2 months of culture (**c**, bar = 0.25 cm); at the end of subculture 2 (6 months) (**d**, bar = 0.9 cm, arrow showing a bulblet); and at the end of subculture 5 (12 months) (**e**, bar = 1.5 cm); **f**: Callus growth under darkness (bar = 1.8 cm)

Seed germination was recorded on all media in varying percentages (36.9 to 91), the highest (91%) on MSBM supplemented with BA (1 mg L^−1^) + NAA (0.4 mg L^−1^) (Table 1), (Fig. 2a). One month old seedlings transferred to glass bottles containing hormone free MSBM, after 3 months of incubation showed 54.8% survival rate. Bulblet formation and callus induction was observed in 37.5% and 19.2% seedlings at its basal ends, respectively (Table 2, Fig. 2b). Out of total 208, a majority of seedlings after 3 months of culture developed 1–2 bulblets, while some seedlings developed a much higher number (8–14 bulblets/seedling). Since only 37.5% seedlings showed development of bulblets on seed germination medium, it was decided to optimize strength of nutrient supply (1X and 1/2 X of MSBM) and different sucrose concentrations in the culture medium. Seedlings on these media survived in varying percentages and showed variation in bulblet formation (Table 3). The maximum seedlings (91.4%) survived on 1/2 X MSBM supplemented with 2.5% sucrose, and in this medium all the seedlings showed at least one bulblet formation (Table 3). Also, 17.1% seedlings showed callus formation on this medium composition (Table 3). Therefore, further experiments for bulblet regeneration and proliferation were carried out on 1/2 X MSBM with 2.5% sucrose.

**Table 1:** Influence of BA and NAA concentrations on seed germination in *F. cirrhosa*

Plant growth regulator (mg L^−1^)		Germination (%)^1^
BA	NAA	
0	0	60.0^c1^
0	0.2	81.3^a^
0	0.4	36.9^d^
1	0	37.7^d^
1	0.2	42.5^d^
1	0.4	91.0^a^
2	0	63.3^bc^
2	0.2	74.4^abc^
2	0.4	60.0^c^

MS salts and vitamins (MSBM) was supplemented with 3% sucrose, 0.4% GPP. Petri-dishes with seeds were incubated at 20 ℃. Each treatment had 120 seeds. Data were recorded after 20 days of culture. Before germination experiment, seeds of *F. cirrhosa* were stored at 4 ℃

^1^Means followed by the different letter of a column are significantly different at 5% level by least significant difference (LSD) test

**Table 2:** Further development of in vitro seedlings (1 month old) on transfer to fresh medium

	%
Survival (%)	54.8
Callus formation (%)	19.2
Bulblet formation (%)	37.5

MS salts and vitamins (MSBM) was supplemented with 3% sucrose, 0.4% GPP. Seedlings were incubated at 20 °C. Data were recorded after 3 months of culture. Total No. of seedlings transferred = 208

**Table 3:** Effects of different salt and sucrose concentrations on growth parameters of 1 month old in vitro seedlings of *F. cirrhosa*

MSBM strength^a^	Sucrose conc. (%)	No. of seedlings inoculated	Survival (%)	Seedlings with bulblet formation (%)	Seedlings with callus formation (%)
1/2 X	0.5	43	72.1	67.4	4.7
1/2 X	1	51	52.9	52.9	0.0
1/2 X	1.5	25	60.0	44.0	24.0
1/2 X	2	47	72.3	68.1	12.8
1/2 X	2.5	35	91.4	91.4	17.1
1/2 X	3	24	75.0	75.0	4.2
1 X	0.5	30	56.7	53.3	6.7
1 X	1	39	69.2	69.2	7.7
1 X	1.5	41	56.1	56.1	12.2
1 X	2	29	34.5	31.0	6.9
1 X	2.5	32	65.6	62.5	6.3
1 X	3	89	51.7	49.4	11.24

^a^MS salts and vitamins (MSBM) were supplemented with 0.4% GPP. Seedlings were incubated at 20 ℃. Data were recorded after 3 months of culture

**Fig. 3:**
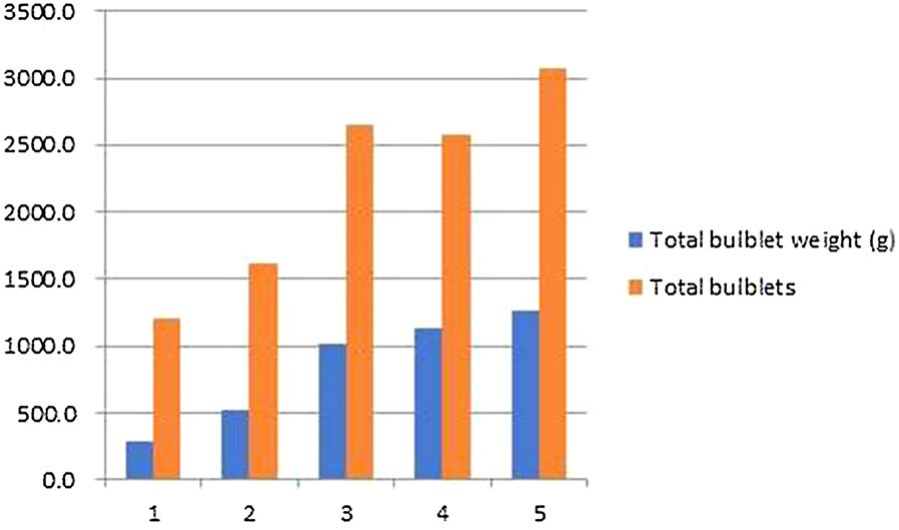
*F. cirrhosa*: Total number of bulblets and weight (g) after each subculture (at 2 months interval)

Four different sections/incisions (longitudinal sections; transverse sections; longitudinal incision; transverse incision) of bulblets cultured on liquid or semi-solid media resulted in varying percentages of survival, and number of bulblets developed (Table 4). Overall, semi-solid media gelled with GPP resulted in higher survival percentages of explants and cent per cent surviving explants showed development of bulblets. However, the number of bulblets varied from 7.2 to 26.7 depending upon the type of section or incision. Transverse sections (Fig. 1b) resulted in the maximum number of bulblets (26.7) (Fig. 2c), while the minimum number of bulblets (7.2) was recorded on transverse incision (Fig. 1d), (Table 4). Further proliferation of bulblets was achieved on their subculture to fresh 1/2 X MSBM medium supplemented with 2.5% sucrose and 0.4% GPP at an interval of 2 months. Proliferation of bulblets at the end of subculture 2 (6 months), and end of subculture 5 (12 months) have been shown in Fig. 2d, e, respectively. The numbers of bulblets and their total fresh weights at the end of each subculture (1–5) have been shown in (Fig. 3). At the end of the subculture 5, more than 3000 bulblets weighing 1270 g could be produced (Fig. 3).

**Table 4:** Effects of different sections/incisions and solid and liquid medium on bulblet regeneration in *F. cirrhosa*

Medium^1^	Treatment^2^	Explant survival (%)^3^	Explants with bulblet formation (%)^3^	No. of bulblet produced^3^
Liquid	A	25.0^c^	91.7^a1^	6.4^bc^
	B	41.7^bc^	91.7^a^	7.0^bc^
	C	75.0^bc^	91.7^a^	3.9^c^
	D	58.3^abc^	91.7^a^	5.3^bc^
Solid	A	66.7^ab^	100.0^a^	11.5^b^
	B	66.7^ab^	100.0^a^	26.7^a^
	C	91.7^a^	100.0^a^	8.6^bc^
	D	83.3^a^	100.0^a^	7.2^bc^

^1^1/2 X MSBM with 2.5% sucrose and 0.4% GPP. Cultures were incubated at 20 °C. Data were recorded after 2 months of culture. Each treatment had 12 replicates

^2^A: longitudinal sections; B transverse sections; C: longitudinal incision; D: transverse incision

^3^Means followed by the different letter of a column are significantly different at 5% level by LSD test

## Callus proliferation

The callus obtained from in vitro seedlings easily multiplied on 1/2 X MSBM supplemented with 2% sucrose and 0.4% GPP (Fig. 2f). The incubation of callus cultures in light (34 μmol m^−2^ s^−1^) or dark had no significant difference in the quantity of callus. However, there was difference in contents of isosteroidal alkaloids in callus produced in dark or light (Table 5).

**Table 5:** LC-MS/MS analysis of peimissine, verticine and verticinone in different plant materials of *F. cirrhosa*

Plant materials	Peimissine (µg/g)	Verticine (µg/g)	Verticinone (µg/g)
In vitro bulblets (6 months)	67.72	–	–
Callus in light (2 months)	48.31	–	–
Callus in dark (2 months)	36.73	0.22	–
Commercial crude drug	223.25	14.49	28.16

## LC–MS/MS analysis

Differences in quantities of three alkaloids in tissue culture derived materials and commercial crude drug samples have been shown in Table 5. In vitro derived bulblets and callus incubated in light (both 2 months old) showed the presence of peimissine (67.72 µg g^−1^ dw and 48.31 µg g^−1^ dw, respectively) and no verticine and verticinone. Callus incubated in dark showed the presence of reduced peimissine content (36.73 µg g^−1^ dw) and the presence of verticine (0.22 µg g^−1^ dw). Commercial crude drug sample of bulbs showed the presence of all 3 alkaloids. Quantities recorded were as follows: peimissine (223.25 µg g^−1^ dw), verticine (14.49 µg g^−1^ dw), and verticinone (28.26 µg g^−1^ dw). The important observation in LC–MS/MS analysis was that callus showed the presence of significant quantities of two alkaloids. While callus incubated under 16/8-h light and dark cycle with an illumination intensity of 34 μmol m^−2^ s^−1^ showed the presence of peimissine (48.31 µg g^−1^ dw), while the callus incubated under complete dark showed the presence of peimissine (36.71 µg g^−1^) and verticine (0.22 µg g^−1^ dw).

## Discussion

*F. cirrhosa* belongs to the family Liliaceae. In many of the species in this family, embryos within the seeds remain underdeveloped at the time of seed dispersal (Kondo et al. 2006). However, the embryos need to grow to a critical length within the seed before germination can occur. Such a phenomenon for embryo growth before germination has been called morphological dormancy (Nikolaeva 1999). Seeds with underdeveloped embryos may have an additional physiological block to germination; such seeds have been described as having morphophysiological dormancy (Baskin and Baskin 1998). In vitro seed germination of such bulbous plants provides a reliable and alternative way of interrupting the dormancy. Different methods like exposure of seeds to different chilling or warm temperatures (Hilhorst 2011), treatment with phytohormones (Seo et al. 2011; Kizil and Khawar 2014), or alternating incubation temperatures of 4 ℃ and 10 ℃ for variable durations in days have been reported (Kizil and Khawar 2014). In the present study, a higher percentage of *F. cirrhosa* seed germination observed under in vitro culture conditions is an important observation. Perhaps, storage of seeds of *F. cirrhosa* at 4 ℃ before germination, and a supplement of BA and NAA in the culture medium boosted a higher percentage of seed germination. Contrary to the present results, Kizil and Khawar (2014) obtained the maximum seed germination (80%) in *Fritillaria persica* L on MS medium enriched with BAP (2 mg L^−1^) plus IBA (1 mg L^−1^). The other serious concern is that in case of *F. cirrhosa,* growth of seedlings in natural habitats is too weak and the development of bulbs is extremely slow, therefore, commercial scale propagation of *F. cirrhosa* by seed has a limited practical application (Ruan et al. 2017). Therefore, aseptic seedlings as a result of seed germination could be reliable starting material for micropropagation of this medicinal herb.

Since bulbs constitute the main source of isosteroidal alkaloids in *F. cirrhosa*, the higher number of bulblets in some in vitro seedlings is an important result considering that in natural conditions, where mostly one bulb develops in a single seedling in about 1 year time. Further, due to extremely slow growth, bulbs take about 5–6 years to grow into an apparent size (Ruan et al. 2017). Contrary to natural conditions, presence of peimissine was detected in 6 months old in vitro bulblets and 2 months old callus (Table 5).

Out of four sectioning/incision treatments of explants, transverse sections of bulblets had larger cut areas exposed to medium and could be the reason for development of the highest number of new bulblets. Between liquid and semi-solid (gelled) media, though the later resulted in comparatively higher number of new bulblets, however, results on development of bulblets in liquid media are also very important. Because, liquid medium can be used to set up bioreactor, and conditions like culture medium composition, and incubation can be optimized for the development of maximum number of bulblets at a commercial scale.

Paek and Murthy (2002) used both agitated liquid and solid media for plant regeneration of *F. thunbergii,* however, the use of liquid culture medium for *F. cirrhosa* has not been reported previously.

In case of *F. cirrhosa*, callus could be easily induced and multiplied. Quantitatively, there was no significant difference in callus grown under light or complete dark; however, there was significant difference with peimissine and verticine in callus grown in light or dark conditions. Callus incubated under complete dark showed the presence of peimissine and verticine, while callus under light conditions showed the presence of peimissine only. Though quantities of alkaloids in callus are less than in commercial crude drug; these results are important since in contrast to bulblets, callus in *F. cirrhosa* can be produced in large quantities in much shorter time. Further culture and incubation conditions can be optimized so that callus can produce all three isosteroidal alkaloids in enhanced quantities. Also, since callus can be induced and multiplied easily on medium free of growth regulators in controlled laboratory conditions, it could be a reliable source of isosteroidal alkaloids throughout the year. Also, since callus contained isosteroidal alkaloids, it can be dried and used as a substitute of bulbs in medicinal formulations.

LC–MS/MS analysis showed significant presence of peimissine in in vitro bulblets. Though the quantity of peimissine in tissue culture derived bulblets (TC-bulblets) is much less than the commercial crude sample, TC-bulblets have advantage since these can be produced in laboratory conditions throughout the year, independent of seasons and climatic conditions.

## Conclusions

The study reports efficient methods of in vitro seed germination, regeneration and propagation of bulblets of *F. cirrhosa* to produce some isosteroidal alkaloids in tissue culture-derived bulblets and callus. The regeneration and proliferation of bulblets in liquid medium has potential scale up application in a bioreactor. Callus which can be easily induced and multiplied under in vitro conditions could be a potential alternative source of isosteroidal alkaloids for commercial use. Also, callus of *F. cirrhosa* can be used to develop cell suspension cultures in bioreactors for the production of these isosteroidal alkaloids. Therefore, we not only can reduce our dependence on collections of *F. cirrhosa* from the wild, but also can help in in situ conservation of this medicinally important plant species.

## Methods

### Plant material

Seeds for the study were grown in Nin Jiom Pharmaceutical Company’s farm in China (https://www.nin-jiom.com.tw/quality-detail/green-base/). The company supplied the seed and partially funded this study considering importance of *Fritillaria cirrhosa.* Seeds obtained were stored at 4 ℃ for 3 months before used. Commercial crude drug sample of *F. cirrhosa* was purchased from Healthy Beautiful Biotech, Co. Ltd., Taichung, Taiwan.

### Establishment of aseptic seedlings

Seeds of *F. cirrhosa* were disinfected by washing several times with sterile distilled water, followed by dipping in 70% ethanol (v/v) for 10 s, then immersed in a solution of 0.5% (v/v) sodium hypochlorite containing 1 drop of Tween-20 for 5 min and this step repeated three times as described in our previous report (Chen et al. 2016). Then 3 rinses (5 min each) with sterile distilled water were carried out under aseptic environment in a laminar flow cabinet. These seeds were cultured in pre-sterilized Petri-dishes (90 mm × 15 mm). Each dish contained 20 mL of half strength (1/2 X) Murashige and Skoog’s (1962) salts and vitamins, hereinafter referred as MS basal medium (MSBM) supplemented with 6-benzylaminopurine (BA) (1, 2 mg L^−1^) and α-naphthalene-acetic-acid (NAA) (0.2, 0.4 mg L^−1^) alone or in combinations. Gellan Gum Powder (GPP) (0.4%) purchased from PhytoTechnology Laboratories^®^ was used as a gelling agent and 3% sucrose was supplemented to each medium. The pH of all the media was adjusted to 5.7 ± 0.1, before addition of GPP, and before autoclaved for 15 min under 1.05 kg cm^−1^ at 121 ℃. The Petri-dishes were incubated in a culture room at 20 ± 2 ℃, a light and dark cycle of 16/8-h and an illumination intensity of 34 μmol m^−2^ s^−1^. After 20 days, germination was recorded and seedlings (1 month old) were transferred to glass bottles (650 mL capacity) each containing 100 mL of MSBM supplemented with 3% sucrose and 0.4% GPP for further seedling growth and development of bulblets.

### Optimization of MSBM and sucrose concentrations

Two salt strengths (1X and 1/2 X) of MSBM and six sucrose concentrations (0.5, 1, 1.5, 2.0, 2.5 and 3%) were used to optimize the culture medium (Table 1). Glass bottles (650 mL capacity) each containing 100 mL of 1X or 1/2 X strength MSBM containing six sucrose concentrations (0.5, 1.0, 1.5, 2.0, 2.5, and 3.0%), and 0.4% GPP were used for further seedling growth and development of bulblets.

### Regeneration and proliferation of bulblets

Aseptic bulblets of uniform size derived from in vitro seedling were used for further regeneration and proliferation. In this experiment, bulblets were sectioned by a sharp scalpel in four different ways, in aseptic conditions in a laminar flow cabinet; longitudinal section (Fig. 1a), transverse section (Fig. 1b), longitudinal incision (Fig. 1c), transverse incision (Fig. 1d). These sectioned explants were placed on two media conditions, liquid and semi-solid. The medium optimized in the previous experiment (1/2 X strength MSBM, 2.5% sucrose, 0.4% GPP) was used. The experiment was carried out in sterile conical flasks (250 mL capacity) containing 50 mL medium. Liquid flasks cultures were kept on a shaker at 60 rpm. Incubation conditions for both liquid and semi-solid media were maintained at 20 ± 2 ℃, and a light and dark cycle of 16/8-h, and an illumination intensity of 34 μmol m^−2^ s^−1^.

### Propagation of bulblets in vitro

For further propagation of bulblets, whole explants with developing bulblets from the previous experiment were subcultured to new bottles containing 100 mL of fresh 1/2 X strength MSBM supplemented with 2.5% sucrose and 0.4% GPP). These subcultures continued at an interval of 2 months. At each subculture, the number of bulblets in each bottle was recorded.

### Proliferation of callus

This experiment was carried out to multiply the callus obtained from the basal parts of in vitro seedlings. Callus was isolated from the seedlings and cultured in pre-sterilized petri-dishes (90 mm diameter × 15 mm height). Each dish contained 20 mL of 1/2 X MSBM supplemented with 2% sucrose, 0.4% GPP. The petri-dishes were incubated in a culture room at 20 ± 2 ℃, a light and dark cycle of 16/8-h with illumination intensity of 34 μmol m^−2^ s^−1^ or in complete dark. For further multiplication, callus was subcultured on the fresh medium at an interval of 2 months.

## LC–MS/MS analysis of isosteroidal alkaloids in bulblets, callus and crude drug samples

### Preparation of standard solutions and Fritillaria samples

The procedure for LC–MS/MS analysis of isosteroidal alkaloids in bulblets, callus and crude drug sample was performed as described in our previous report (Yang et al. 2017). The isosteroidal alkaloids standards of peimisine, peiminine, and primine were purchased from the National Institute for Food and Drug Control (Beijing, China). The stock solutions of 1 mg mL^−1^ were prepared separately by using methanol as solvent and stored in dark at minus 30 ℃ in a deep freezer before used. Working solutions were prepared by diluting the stock solutions with 20% methanol in distilled water to an appropriate concentration. The peimisine, peiminine, and primine were extracted from tissue culture materials and commercially available *Fritillaria* by using QuEChERS extraction technique as follows: 1 g of homogenized *Fritillaria* samples (In vitro bulblets, callus, and crude drug separately) and 5 mL of 30% acetonitrile aqueous solution containing 1% ammonium hydroxide were taken in a 15 mL centrifuged tube. After ultrasonic bathing for 30 min, 2.5 g of magnesium sulfate-sodium acetate mixture (4:1, w/w) was added to the sample and the tube was centrifuged for 10 min at 4000 rpm. An aliquot of 2 mL extract was moved to other centrifuging tube and subsequently 15 mg of primary secondary amine (PSA) was added. After centrifuging for 19 min at 14,000 rpm, the supernatant was dried then reconstructed by 1 mL of 20% acetonitrile aqueous solution. A 5 μL extract was injected into the LC–MS/MS for analysis.

### Instrumentation

The analysis was carried out using a Surveyor LC system (ThermoScientific, San Jose, CA, USA) composed of an autosampler and a quaternary pump. Peimisine, peiminine, and primine fractions were separated by using Accucore C_18_ (ThermoSientific) column (2.1 × 150 mm, 2.6 μm) at a room temperature. The mobile phase A and B were purified by water containing 0.05% diethylamine (DEA) and acetonitrile-methanol (9:1, v/v) solution containing 0.05% DEA, respectively. The separating gradient was initialized at 20% phase B held for 1 min at the flow rate of 0.13 mL min^−1^ and subsequently increased linearly to 60% phase B at the flow rate of 0.16 mL min^−1^ in 3 min. Then the gradient increased to 95% phase B at the flow rate of 0.2 mL min^−1^ in 4 min and held for 5 min. In the final step, the gradient was reversed to 20% phase B at flow rate of 0.2 mL min^−1^ and held for 6 min at the flow rate of 0.13 mL min^−1^ for equilibrium. The injection volume was 5 μL. Mass spectrometric analyses were performed on an LTQ linear ion trap tandem mass spectrometer (ThermoScientific) equipped with an electrospray ionization (ESI) interface. The mass spectra of target isosteroidal alkaloids were obtained in positive ionization mode. The optimal ESI ionization conditions were as follow: spray voltage was 4.6 kV; the capillary temperature was 300 ℃; the sheath gas pressure was 35 arbitrary units; the auxiliary gas was 5 arbitrary units. The mass scan was ranged from 300 to 1000 m z^−1^. The protonated molecule of peimisine, peiminine, and primine at 428 m z^−1^, 430 m z^−1^, and 432 m z^−1^ were chosen as extracted ions for quantitative analysis of isosteroidal alkaloids in *Fritillaria* samples, respectively.

## Statistical analysis

Statistical analysis was carried as per the procedure described in our previous report (Chen et al. 2016). Using SAS 9.1 software, data including the number of seeds showing germination (Table 1), number of seedlings survived, showing bulblet formation and callus formation (Table 2, 3), number of explants survived, showing bulblet formation, callus formation, and bulblet length (Table 4) were subjected to the least significant difference (LSD) tested at 5% probability level (p > 0.05) wherever possible. Each treatment had minimum 20 replicates (unless stated). The experiments were repeated three times except LC–MS/MS analysis.

## Data Availability

Data of this study is available with the first author Dr. Chang.

## Abbreviations

BAP: 6-Benzylaminopurine
DEA: Diethylamine
GPP: Gellan gum powder
LC–MS/MS: Liquid chromatography–mass spectrometry
MSBM: Murashige and Skoog’s basal medium (salts and vitamins)
NAA: α-Naphthalene-acetic-acid
V/V: Volume/Volume

## References

[CR1] Baskin CC, Baskin JM (1998). Seeds: ecology, biogeography, and evolution of dormancy and germination.

[CR2] Bharali S, Khan M (2011). Climate change and its impact on biodiversity: some management options for mitigation in Arunachal Pradesh. Curr Sci.

[CR3] Carasso V, Hay FR, Probert RJ, Mucciarelli M (2011). Temperature control of seed germination in *Fritillaria tubiformis* subsp. moggridgei (Liliaceae) a rare endemic of the South-west Alps. Seed Sci Res.

[CR4] Chen XQ, Mordak HV, Wu ZY, Peter PH (2000). *Fritillaria* Linnaeus. Flora of China.

[CR5] Chen CC, Agrawal DC, Lee MR, Lee RJ, Kuo CL, Wu CR, Tsay HS, Chang HC (2016). Influence of LED light spectra on in vitro somatic embryogenesis and LC–MS analysis of chlorogenic acid and rutin in *Peucedanum japonicum* Thunb: a medicinal herb. Bot Stud..

[CR6] Cunningham AB, Brinckmannc JA, Peid SJ, Luo P, Schippmannf U, Long X, Bi YF (2018). High altitude species, high profits: can the trade in wild harvested *Fritillaria cirrhosa* (Liliaceae) be sustained?. J Ethnopharmacol.

[CR7] Ding K, Lin G, Ho YP, Cheng TY, Li P (1996). Prederivatization and high-performance liquid chromatographic analysis of alkaloids of bulbs of *Fritillaria*. J Pharmaceut Sci.

[CR8] Gao SL, Zhu DN, Cai ZH, Jiang Y, Xu DR (1999). Organ culture of a precious Chinese medicinal plant–*Fritillaria unibracteata*. Plant Cell Tiss Organ Cult.

[CR9] Hilhorst HW (2011). Standardizing seed dormancy research. Methods Mol Biol.

[CR10] Kizil S, Khawar KM (2014). The effects of plant growth regulators and incubation temperatures on germination and bulb formation of *Fritillaria persica* L. Propag Ornament Plants.

[CR11] Kondo T, Sato C, Baskin JM, Baskin CC (2006). Post-dispersal embryo development, germination phenology, and seed dormancy in *Cardiocrinum cordatum* var. glehnii (Liliaceae s. str.), a perennial herb of the broadleaved deciduous forest in Japan. Am J Bot.

[CR12] Li HJ, Jiang Y, Li P (2006). Chemistry, bioactivity and geographical diversity of steroidal alkaloids from the Liliaceae family. Natur Prod Rep.

[CR13] Li K, Wu W, Zheng Y, Dai Y, Xiang L, Liao K (2009). Genetic diversity of *Fritillaria* from Sichuan province based on ISSR. China J Chin Materia Medica.

[CR14] Lin G, Li P, Li SL, Chan SW (2001). Chromatographic analysis of *Fritillaria* isosteroidal alkaloids, the active ingredients of Beimu, the antitussive traditional Chinese medicinal herb. J Chromatogr A.

[CR15] Murashige T, Skoog F (1962). A revised medium for rapid growth and bioassays with tobacco culture. Physiol Plant.

[CR16] Nikolaeva MG (1999). Patterns of seed dormancy, germination as related to plant phylogeny and ecological and geographical conditions of their habitats. Russ J Plant Physiol.

[CR17] Özcan S, Parmaksiz I, Mirici S, Çöçü S, Uranbey S, İpek A, Sancak C, Sarihan E, Gürbüz B, Sevimay C, Arslan N, Xu (2007). Efficient in vitro bulblet regeneration from immature embryos of endemic and endangered geophyte species in *Sternbergia*, *Muscari* and *Fritillaria* genera. Biotechnology and Sustainable Agriculture 2006 and Beyond.

[CR18] Paek KY, Murthy HN (2002). High frequency of bulblet regeneration from bulb scale sections of *Fritillaria thunbergii*. Plant Cell Tiss Organ Cult.

[CR19] Petric M, Subotic A, Trifunovic M, Jevremovic S (2012). Morphogenesis in vitro of *Fritillaria* species. Floricult Ornament Biotech.

[CR20] Pyakurel D, Baniya A (2011). NTFPs: impetus for conservation and livelihood support in Nepal. A reference book on ecology, conservation, product development and economic analysis of selected NTFPs of Langtang area in the Sacred Himalayan Landscape.

[CR21] Ruan HL, Zhang YH, Wu JZ (2002). Advances in studies on non-alkaloid constituents of *Fritillaria* L. plants. Chin Trad Herbal Drugs.

[CR22] Ruan X, Cui WX, Yang L, Li ZH, Liu B, Wang Q (2017). Extraction of total alkaloids, peimine and peiminine from the flower of *Fritillaria thunbergii* Miq using supercritical carbon dioxide. J Utilization.

[CR23] Seo M, Jikumaru Y, Kamiya Y, Kermode AR (2011). Profiling of hormones and related metabolites in seed dormancy and germination studies. Seed Dormancy Methods and Protocols.

[CR24] The State Pharmacopoeia Commission of P.R. China (2015) Pharmacopoeia of the People’s Republic of China 2015. China

[CR25] Wang YH, Dai Y, He ZS, Sun YX, Yan SJ, Xu SJ, Wang XR (2010). The effects of in vitro culture conditions on regeneration of *Fritillaria cirrhosa*. Zhong Yao Cai..

[CR26] Wang YH, Dai Y, Yan SJ, Sun YX, He ZS, Xu SJ, Wang XR (2010). Study on fast proliferation conditions of *Fritillaria cirrhosa* D. Don callus. South-West China J Agri Sci.

[CR27] Yang CL, Chang HC, Chen CY, Lee MR (2017). Determination of isosteroidal alkaloids in *Fritillaria* spp. by LC-MS/MS. J Chin Mass Spectrometry Soc.

[CR28] Zhang D, Gao L, Yang Y (2010). Genetic diversity and structure of a traditional Chinese medicinal plant species, *Fritillaria cirrhosa* (Liliaceae) in southwest China and implications for its conservation. Biochem Syst Ecol.

